# Some common deleterious mutations are shared in SARS-CoV-2 genomes from deceased COVID-19 patients across continents

**DOI:** 10.1038/s41598-023-45517-1

**Published:** 2023-10-30

**Authors:** Md. Aminul Islam, Abdullah Al Marzan, Md. Sakil Arman, Shatila Shahi, Tahsin Islam Sakif, Maqsud Hossain, Tofazzal Islam, M. Nazmul Hoque

**Affiliations:** 1 Advanced Molecular Lab, Department of Microbiology, President Abdul Hamid Medical College, Karimganj, Kishoreganj 2310 Bangladesh; 2 COVID-19 Diagnostic Lab, Department of Microbiology, Noakhali Science and Technology University, Noakhali, 3814 Bangladesh; 3 Department of Biochemistry and Molecular Biology, Shahjalal University of Science and Technology, Sylhet, 3114 Bangladesh; 4 Lane Department of Computer Science and Electrical Engineering, West Virginia University, Morgantown, WV 26506-6109 USA; 5 University of Nottingham, Sutton Bonington Campus, LE12 5RD, Loughborough, NG7 2RD Leicestershire UK; 6 Institute of Biotechnology and Genetic Engineering (IBGE), Bangabandhu Sheikh Mujibur Rahman Agricultural University, Gazipur, 1706 Bangladesh; 7 Molecular Biology and Bioinformatics Laboratory, Department of Gynecology, Obstetrics and Reproductive Health, Bangabandhu Sheikh Mujibur Rahman Agricultural University, Gazipur, 1706 Bangladesh

**Keywords:** Computational biology and bioinformatics, Evolution, Genetics, Microbiology, Molecular biology, Diseases

## Abstract

The identification of deleterious mutations in different variants of SARS-CoV-2 and their roles in the morbidity of COVID-19 patients has yet to be thoroughly investigated. To unravel the spectrum of mutations and their effects within SARS-CoV-2 genomes, we analyzed 5,724 complete genomes from deceased COVID-19 patients sourced from the GISAID database. This analysis was conducted using the Nextstrain platform, applying a generalized time-reversible model for evolutionary phylogeny. These genomes were compared to the reference strain (hCoV-19/Wuhan/WIV04/2019) using MAFFT v7.470. Our findings revealed that SARS-CoV-2 genomes from deceased individuals belonged to 21 Nextstrain clades, with clade 20I (Alpha variant) being the most predominant, followed by clade 20H (Beta variant) and clade 20J (Gamma variant). The majority of SARS-CoV-2 genomes from deceased patients (33.4%) were sequenced in North America, while the lowest percentage (0.98%) came from Africa. The ‘G’ clade was dominant in the SARS-CoV-2 genomes of Asian, African, and North American regions, while the ‘GRY’ clade prevailed in Europe. In our analysis, we identified 35,799 nucleotide (NT) mutations throughout the genome, with the highest frequency (11,402 occurrences) found in the spike protein. Notably, we observed 4150 point-specific amino acid (AA) mutations in SARS-CoV-2 genomes, with D614G (20%) and N501Y (14%) identified as the top two deleterious mutations in the spike protein on a global scale. Furthermore, we detected five common deleterious AA mutations, including G18V, W45S, I33T, P30L, and Q418H, which play a key role in defining each clade of SARS-CoV-2. Our novel findings hold potential value for genomic surveillance, enabling the monitoring of the evolving pattern of SARS-CoV-2 infection, its emerging variants, and their impact on the development of effective vaccination and control strategies.

## Introduction

The emergence and rapid spread of coronavirus disease 2019 (COVID-19), caused by severe acute respiratory syndrome coronavirus-2 (SARS-CoV-2), a potentially fatal disease, swiftly resulted in public health crises worldwide^1, 2^. The World Health Organization (WHO) declared COVID-19 as a Public Health Emergency of International Concern due to its disastrous impact on global systems, including public health, the economy, and communities. This declaration was prompted by widespread infection, dynamic mutations, and the emergence of new variants^3, 4^. Since its emergence in 2019, SARS-CoV-2 has infected more than 697 million individuals, resulting in approximately 6.9 million deaths worldwide as of October 2023 (https://www.worldometers.info/coronavirus/). While highly dynamic mutations in SARS-CoV-2 are a topic of concern, understanding the frequency and distribution of these mutations in emerging variants may be crucial for formulating an effective strategy to curb the spreading of infection^2, 5, 6^.

The SARS-CoV-2 belongs to the *Betacoronavirus* genus within the Coronaviridae family and is a positive-sense, single-stranded RNA (+ssRNA) virus (Islam et al.^33^). Approximately 30 kilobase-sized genome of the novel SARS-CoV-2 encodes several smaller open reading frames (ORFs)^7, 8^. SARS-CoV-2 genome encodes seven accessory proteins (ORFs), for example ORF3a, including ORF3a, ORF3b, ORF6, ORF7a, ORF7b, ORF8, and ORF9b, which are thought to play a role in immune evasion^9, 10^. Additionally, there is evidence that the SARS-CoV-2 genome encodes two more accessory proteins, ORF9c and ORF10, both of which have been suggested to play roles in the immune evasion process^9, 10^. These ORFs encode for different proteins, for example, the replicase polyprotein, spike (S) glycoprotein, envelope (E), membrane (M), nucleocapsid (N) proteins, accessory proteins, and other non-structural proteins (nsp)^8, 11^. In addition, N proteins are embedded in the main genome architecture along some accessory proteins 3a, 3b, 7a, 8b, 9b, and 10^12, 13^. Within a few months of its emergence, several more transmissible variants of SARS-CoV-2 have been detected worldwide, potentially evading natural and vaccine-induced immunity and resulting in an increased rate of SARS-CoV-2 infections compared to its ancestral strains^14, 15^. The SARS-CoV-2 underwent a mutation rate of about (1.19–1.31) × 10^−3^/site/year^16^. SARS-CoV-2 genomes from humans reported to share more than 90% identity with bat-derived (e.g., BatCoV RaTG13)^17^ and pangolin-derived^18^ coronaviruses. SARS-CoV-2 interacts with human angiotensin-converting enzyme 2 (hACE2) for entry, which is expressed in various human organs^17^. A recent study provided evidence that ACE2 from various animals, including monkeys, rabbits, pangolins, horses, cats, foxes, dogs, pigs, wild Bactrian camels, bovines, goats, and sheep, can bind to the SARS-CoV-2 S protein receptor binding domain (RBD) similarly to hACE2^19^. Therefore, SARS-CoV-2 may also exploit ACE2 from other animals as a receptor to infect these animals^20^. For instance, dog ACE2 (dACE2) has been reported to bind to the RBD of SARS-CoV-2^20^. However, few important mutations in the RBD binding interface play a pivotal role in the binding affinity of RBD to both dACE2 and hACE2^20^. While the majority of the mutations have a slight effect to none on the transmission and progression of SARS-CoV-2 infection^21^, a few spike protein mutations are responsible for rapid transmissibility, vaccine immune evasion, severe pathogenicity^21, 22^. Till now, the SARS-CoV-2 has evolved through several novel mutations, generating dangerous variants, including Alpha (B.1.1.7) containing seven novel mutations in the spike protein, Beta (B.1.351) with 9, Gamma (P.1) with 12, and Delta (B.1.6, B.1.6.2) with 17 mutations^2, 23^. RNA viruses like SARS-CoV-2 continuously evolve as changes in the genetic code (caused by genetic mutations or viral recombination) occur during genome replication. Based on the potential threats of these viral variants in terms of transmission, disease severity, immunocompromise, etc., the Center for Disease Control and Prevention (CDC) has classified SARS-CoV-2 variants into different categories, Variant of Interest (VOI), Variant of Concern (VOC), Variants Being Monitored (VBM) and Variant of High Consequences (VOHC)^24^. Although a significant number of SARS-CoV-2 variants have emerged during the COVID-19 pandemic, the Omicron variant (B.1.1.529) has now become the dominant VOC^25^. Several studies reported that SARS-CoV-2 mutations are directly or indirectly associated with COVID-19 severity^2^. It was reported that mutation in the P25L site of the non-structural Orf3a region involving antigen diversity and epitope loss of immune B cells is associated with a higher death rate^23^. Similarly, RNA-dependent RNA polymerase (RdRp) mutation in the NSP12 region increased disease severity. Higher mutational frequencies were also identified in ORF1a, S, and N genes of SARS-CoV-2^26^. The reported studies have yielded good evidence for viral transmissibility that all VOCs are more transmissible than the wild-type virus^27^. Variant escape from antibody neutralization can reduce the effectiveness of vaccination programs and necessitate the development of modified vaccines or administration of booster doses^28^. Furthermore, tracking the emergence of new variants and/or clades of SARS-CoV-2 is one of the top priorities throughout the globe.

Genomic alterations, such as amino acid substitutions in the SARS-CoV-2 genome, may be associated with viral evolution, changes in biological properties, and virulence^29^. Several earlier studies reported that mutation frequency in SARS-CoV-2 genomes might vary according to geographic areas and environmental factors^8, 30^. Thus, it is crucial to find out SARS-CoV-2 mutations and their patterns, and frequency in deceased COVID-19 people across the globe, which will help preempt the occurrence of VOIs and VOCs. Mutations and host modulation are recognized as the primary contributors to COVID-19 pathogenesis. Substituting AA residues in the epitope region renders antibody-mediated immunity and increases virus replication^31^. There have been only a few studies, including one from India^32^, that have reported mutations in SARS-CoV-2 genomes from COVID-19 deceased patients since its emergence in December 2019. This study aims to investigate the mutational patterns within SARS-CoV-2 genomes from deceased COVID-19 patients worldwide, spanning various demographic groups. Additionally, we seek to provide preliminary insights into the mutation patterns and whether these changes are influenced by environmental and genetic factors in the host. Using a robust phylogenetic tree, we endeavor to trace the global spread of these variants, offering insights into the evolutionary pressures within SARS-CoV-2 genomes, which may impact virulence and the outcome of COVID-19 cases. The findings of this research shed light on mutational patterns in SARS-CoV-2 genomes associated with the mortality of COVID-19 patients across continents.

## Results

### Demographic summary of the retrieved genomes of the SARS-CoV-2

**Figure 1:**
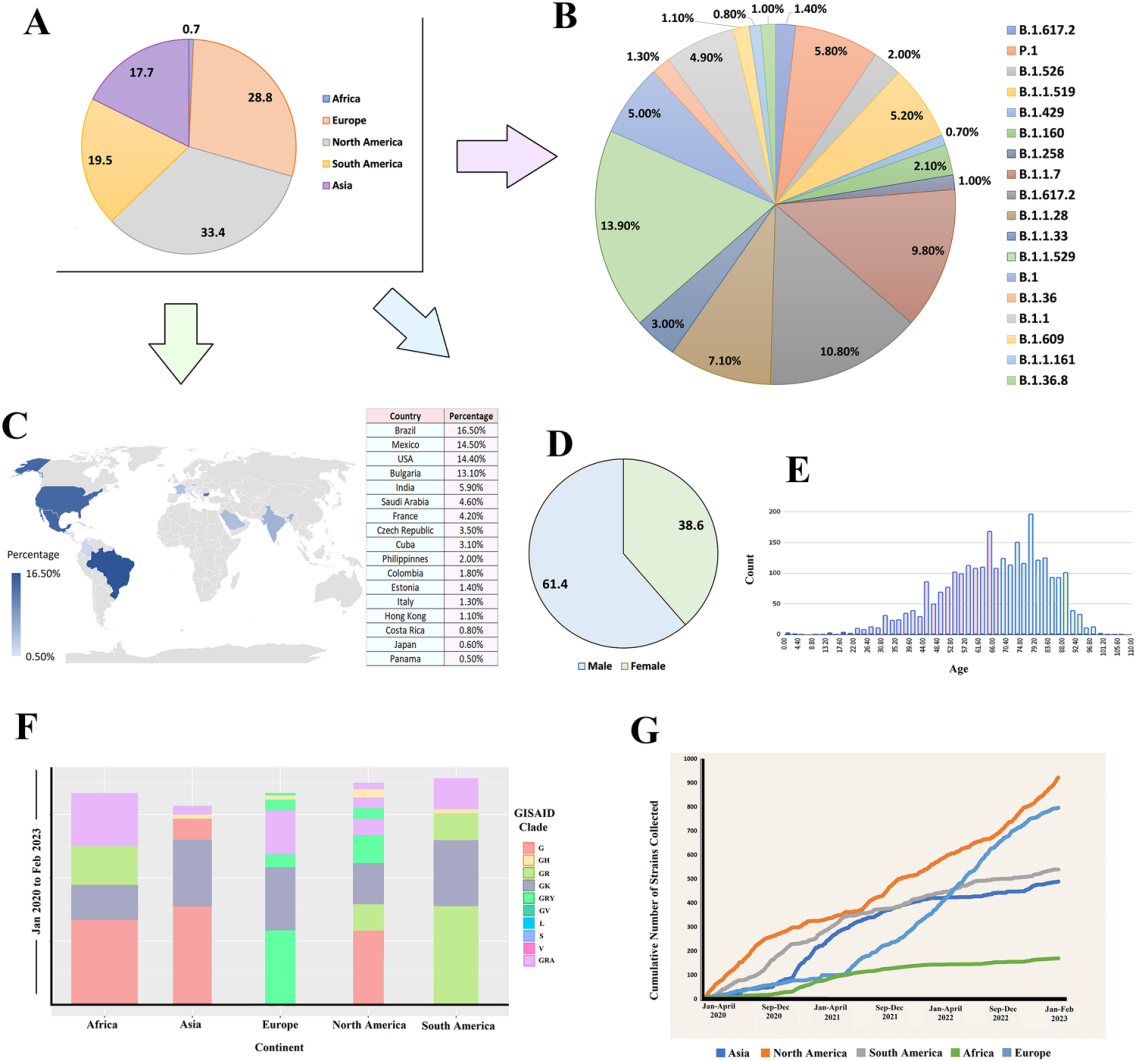
Retrieved SARS-COV-2 whole genome sequences (WGS) obtained from deceased COVID=19 patients worldwide from the global initiative on sharing all influenza database (GISAID). (**A**) SARS-CoV-2 genomes submitted to the GISAID from five continents of the globe between January 2020 and February 2023. (**B**) Distribution of sequences throughout several lineages during the time span. (**C**) SARS-CoV-2 genomes sequenced by different countries from deceased patients. (**D**) Gender-wise (male and female) and (**E**) age-wise distribution of the selected sequences. (**F**) Distribution of different clades in five continents. (**G**) Cumulative number of strains retrieved during the time frame from five continents.

To investigate the spectrum of nucleotide (NT) and amino acid (AA) mutations and their effects  in different variants of the SARS-CoV-2, sequenced from COVID-19 deceased patients, we retrieved 243,270 whole genome sequence (WGS) with high read coverage (> 29,000 bp) from the global initiative on sharing all influenza data (GISAID) up to February 2023. After a thorough filtering of these genomes, 5724 complete genomes belonged to COVID-19 deceased patients from different demographics were selected for further analysis. These WGS data (n = 5724) comprised SARS-CoV-2 genome sequences from 123 countries and five continents (e.g., Asia, Africa, Europe, North America and South America) of the globe. The geographical distribution of the SARS-CoV-2 WGS from deceased COVID-19 people reveals that 33.4% of  the genomes were sequenced from North America followed by 28.8% from Europe, 19.9% from South America, 17.7% from Africa, and 0.7% from Asia (Fig. 1A).

We found B.1.1.529 (Omicron) as the most prevalent (13.90%) among all the lineages detected whereas B.1.617.2 (Delta) and B.1.1.7 (Alpha) were also significantly prevalent; comprising 10.80% and 9.80% of the sequences, respectively (Fig. 1B). However, B.1.1.28 (Brazilian variant), P.1 (Gamma), and B.1.1.519 (Mexican variant) were also observed to be predominant in 7.10, 5.80 and 5.00% of the SARS-CoV-2 genomes, respectively (Fig. 1B). By comparing these data according to different countries of origin, we found that Brazil contributed the highest (16.5%) amount of SARS-CoV-2 WGS data sequenced from deceased COVID-19 patients, followed by Mexico (14.5%), the USA (14.4%), Bulgaria (13.1%), and India (5.1%). The lowest amount of WGS (0.5%) was sequenced from deceased COVID-19 patients in Panama (Fig. 1C). According to the metadata, 61.4% of deceased COVID-19 patients were male while 38.6% were female (Fig. 1D) with an average age of approximately 70 years (Fig. 1E), which was not explicitly stated in the GISAID. Further analysis demonstrated that male patients with an average age of 55 years had higher COVID-19 risk than female patients.

The “G” clade of the SARS-CoV-2 was found to be predominated in the deceased COVID-19 patients of the Asian, African and North American regions while most of the death cases in Europe were registered with “GRY” clade. In contrast, most death cases were registered for “GR” clade in the South American continent (Fig. 1F). By looking at the cumulative death cases registered throughout study period (from January 2020 to February 2023), we found that most of the WGS data (approximately 1000 sequences) from deceased COVID-19 patients were sequenced from the North American residents. Though all the countries had submitted their WGS irrespective of regional barrier, African regions are found as the lowest possible data generating zone from deceased COVID-19 patients (less than 200 sequences) (Fig. 1G). Relevant demographic and medical data are described in Data S1.

### Phylogenetic diversity of the SARS-CoV-2 genomes of the deceased COVID-19 patients

**Figure 2:**
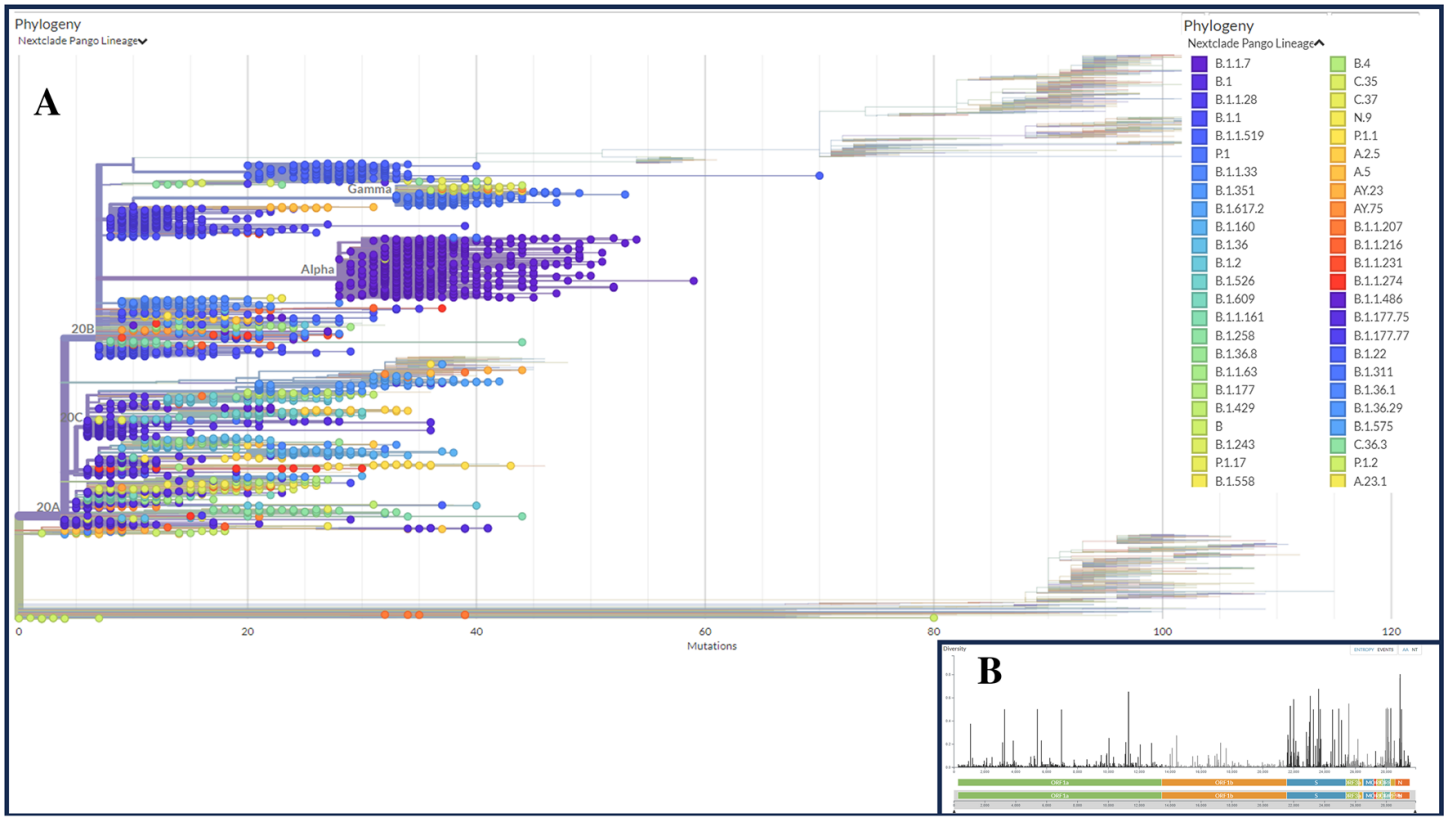
Phylogenetic analyses of the 5,724 SARS-CoV-2 genomes sequenced from the COVID-19 deceased patients worldwide. (**A**) A detailed phylogenetic tree presenting all the significant clades associated with deceased COVID-19 patients. (**B**) Value of the entropy change (distribution of mutational frequency overall the SARS-CoV-2 genome) throughout the SARS-CoV-2 genome based on mutation count for each position. The maximum-likelihood tree was generated using Nextclade Web 2.14.1 web-based tool (https://clades.nextstrain.org/results; accessed on October 10, 2023).

To determine phylogenetic characteristics of the SARS-CoV-2 genomes of the deceased COVID-19 patients, we built a maximum likelihood (ML) tree based on aligned full length sequences using Nextclade Web 2.14.1 web-based tool (https://clades.nextstrain.org/) (Fig. 2, Data S2). The WGS data assembled from deceased COVID-19 patients around the world showed the formation of 21 Nextstrain clades, including four VOCs: 20I (alpha, V1), 20H (Beta, V2), 20J (Gamma, V3), and 21A, 21I, and 21J (Delta V4). In addition, the study genomes belonged to the VOIs, such as 21C (Epsilon), 21G (Lambda), and 21H (Mu), other assigned and unassigned clades including 21B (Kappa), 21F (Lota), 20E (EU1), 19A, 19B, 20A, 20B, 20C, 20D, and 20G (Fig. 2A). The viral clade distribution of the study genomes represented some of the SARS-CoV-2 genetic clades that were circulating worldwide during January 2020 to February 2023. We further explored the diversity of SARS-CoV-2 genomes by comparing the distance matrix of the SARS-CoV-2 strains of the deceased patients to the Wuhan-Hu-1/NC 045512 reference strain. Nextstrain classification revealed that clade 20I (Alpha, V1) was prevalent in 20% of the study genomes, whereas clade 20H (Beta, V2) and 20J (Gamma, V3) were found to be prevalent in 2.42% and 6.33% genomes, respectively (Fig. 2B). Moreover, clade 21A, 21I, and 21J of the Delta variant (B.1.617.2) accounted for 1.48% of the study genomes while clade 21F of the Lota variant was represented by 1.45% genomes. However, only four SARS-CoV-2 genomes in our investigation belonged to the Kappa variant (B.1.617.1), which was prevalent in India and possessed three significant alterations at the sites of L452R, E484Q, and P681R. In addition, 25, 3, and 16 sequences were belonged to Epsilon (21C), Lambda (21G), and Mu (21H), respectively, which are labeled as “VOI.” The most prevalent clades in our analysis were 20B (30%) followed by 20A (22.0%). In this analysis, the most mutational frequency was observed in the spike protein region followed by ORF1a fragment. Whereas the least changes were observed in ORF1b region. The highest peak was observed in the N portion (Fig. 2B).

### Frequency and distribution of nucleotide mutations in SARS-CoV-2 genomes of the deceased COVID-19 patients

**Figure 3:**
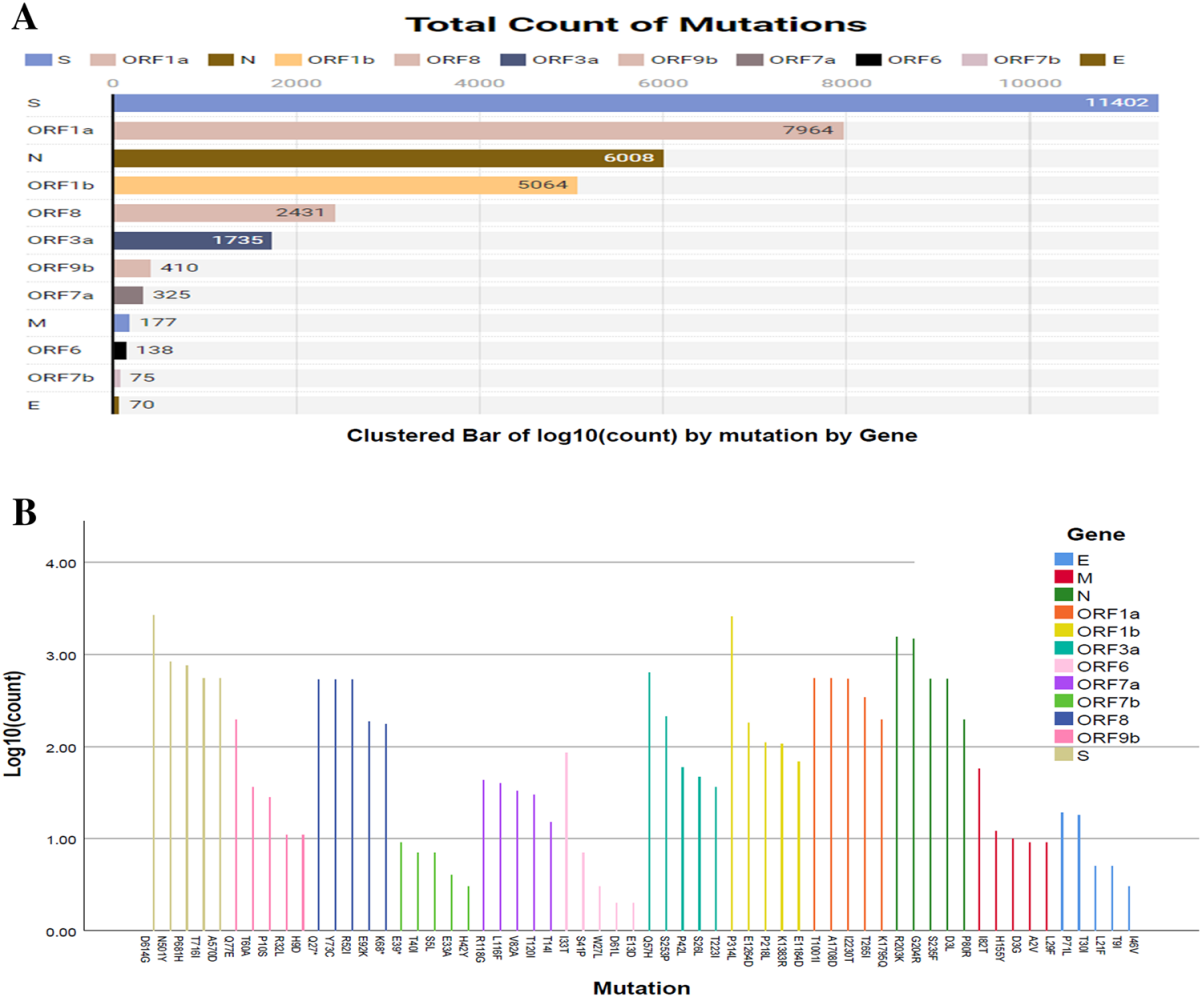
The frequency of nucleotide (NT) mutations found throughout the SARS-CoV-2 genomes of the deceased COVID-19 patients. (**A**) The number of conversions respective to specific genes or segments of the SARS-CoV-2 genome. (**B**) The maximum frequency of NT mutations in particular region of  the SARS-CoV-2 genome. In both cases, specific gene regions were colored with frequency.

To determine the frequency and distribution of nucleotide (NT) mutations, we further analyzed 5,724 SARS-CoV-2 genomes from deceased COVID-19 patients of diverse demographics. We detected an average of 12.9 NT mutations per genome. The overall NT mutation frequencies in the SARS-CoV-2 genomes sequenced from deceased COVID-19 patients are shown in Fig. 3. Our comprehensive mutational analysis identified 35,799 NT mutations across the entire dataset of 5724 SARS-CoV-2 genomes, of which 11,402 (highest) NT mutations were solely found in the *S* gene. Conversely, the *E* gene possessed the lowest number (n = 94) of NT mutations (Fig. 3A). In addition, the number of NT mutations in ORF1a, N, ORF1b, ORF8, ORF3a, ORF9b, ORF7a, M, ORF6, ORF7b, and E segments were 7964, 6008, 5064, 2431, 1735, 410, 325, 177, 138, 75 and 70, respectively (Fig. 3A). The highest number of NT mutations were identified in D614G positions, followed by N501Y, P681H, T716I, and A570D in the spike (S) protein (Fig. 3B). Of the identified NT mutations, the four most frequent NT mutations such as T1001I, A170BD, I2230T, and T265I were identified in the *N* gene. Herein this study, we spotted three most NT mutations (e.g., P314L, E1264D, and P218L) in the *ORF1b* gene. In addition, *E*, *M*, *ORF3a*, *ORF6a*, *ORF7b*, *ORF8* and *ORF9b* genes possessed the highest number of NT mutations at P71L, I82T, Q57H, I33T, T4OI, Y73C and Q77E positions, respectively. Other genes with significant NT mutation frequencies at specific sites included P71L (*E*-gene), T4OI (0RF7b), I33T (ORF6a), I82T (M), T40I (ORF7b), Q77E (ORF9b), Q57H (ORF3a) and Y73C (ORF8) (Fig. 3B).

Another notable finding of this study is the prediction of the NT alterations in the SARS-CoV-2 genomes of various nations.  We compared the NT mutational spectra in top 12 countries (Table 1), where the highest number of COVID-19 associated deaths were reported. The frequency of the NT mutations at D614G position in the *S* gene was prominent in 12 nations with the most significant incidence of COVID-19 deaths (Table 1). Similarly, the maximum number of NT mutations were identified in T1001I, G204R, E1264D, P314L, E92K, Q57H, Q77E, S5L, L29F, S41P, and T40I positions of SARS-CoV-2 genomes. The SARS-CoV-2 genomes sequenced from deceased COVID-19 patients of the USA showed maximum NT mutations at I2230T position in *ORF1a*, D3L in *N*, P218L in *ORF1b*, Y73C in *ORF8*, Q57H in *ORF3a*, P10S in *ORF9b*, A43S in *ORF7a*, T175M in *M*, E13D in *ORF6*, T40I in *ORF7b*, and P71L in *E* genes. In contrast, genomes sequenced from India showed the highest NT mutation frequency at D614G in *S* gene followed by T1001I in *ORF1a*, G204R in *N* gene, K1383R in *ORF1b*, R52I in *ORF8*, P42L in *ORF3a*, Q77E in *ORF9b*, N38T in *ORF7a*, L87F in *M* gene, I14T in *ORF6*, S5L in *ORF7b* and T9I in *E* gene (Table 1). These findings imply that while some SARS-CoV-2 NT mutations were responsible for its evolution, a few may benefit viral adaptation in a specific demographic distribution. Variations in NT mutation patterns in SARS-CoV-2 genomes may be attributable to population age distribution, gender, host immunity, and socioeconomic level.

**Table 1:** The nucleotide (nNT) mutations with the highest frequency predicted at various loci of SARS-CoV-2 genomes extracted from deceased COVID-19 patients of different countries.

Country	Loci of SARS-CoV-2 genome											
	S	ORF1a	N	ORF1b	ORF8	ORF3a	ORF9b	ORF7a	M	ORF6	ORF7b	E
Brazil	D614G	T1001I	D3L	E1264D	E92K	Q57H	Q77E	T40I	L29F	S41P	S5L	P71L
Mexico	D614G	A1708D	R203K	P314L	R52I	P42L	P10S	D36Y	A104V	S43P	T40I	T30I
USA	D614G	I2230T	D3L	P218L	Y73C	Q57H	P10S	A43S	T175M	E13D	T40I	P71L
India	D614G	T1001I	G204R	K1383R	R52I	P42L	Q77E	N38T	L87F	I14T	S5L	T9I
Iran	D614G	I2230T	R203K	E1264D	E92K	Q57H	P10S	T40I	A69S	P57L	E33A	P71S
Spain	N501Y	T1001I	G204R	P218L	R52I	S253P	R32L	M1K	V70F	K42N	H42Y	V49L
China	D614G	T1001I	P80R	E1264D	E92K	P42L	Q77E	A15S	A69S	S41P	S5L	R69G
UK	D614G	A1708D	R203K	K1383R	E92K	Q57H	R32L	S5L	L29F	W27L	T40I	T30I
France	D614G	I2230T	G204R	P218L	R52I	P42L	T60A	A15S	S4F	D61L	S5L	P71L
Italy	N501Y	T1001I	S235F	K1383R	E92K	S253P	P10S	V21F	H125Y	L4V	T40I	T30I
Japan	D614G	A1708D	P80R	P314L	Y73C	Q57H	Q77E	S5L	L29F	S41P	S5L	T30I
Germany	D614G	T1001I	G204R	P314L	Y73C	S253P	P10S	S5L	T175M	S43P	T40I	T30I
Highest	D614G	T1001I	G204R	E1264D, P314L	E92K	Q57H	Q77E	S5L	L29F	S41P	T40I	P71L

### Point-specific amino-acid mutations in SARS-CoV-2 genomes of the deceased COVID-19 patients

**Figure 4:**
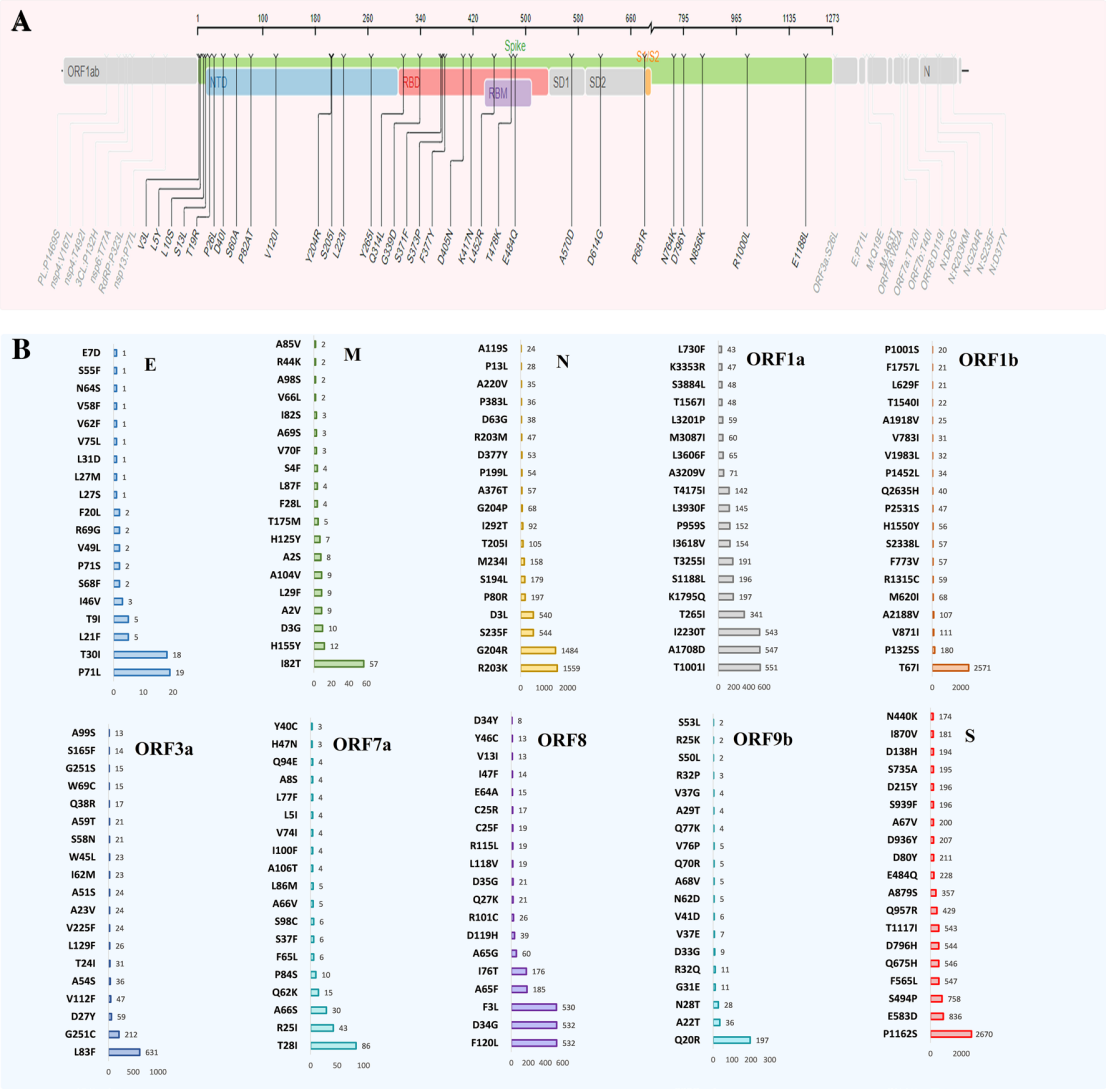
Genomic deletion analysis in SARS-CoV-2 whole genome sequences of the deceased COVID-19 patients. (**A**) Mapping of amino acid (AA) mutations in the spike (S) glycoprotein of SARS-CoV-2 genome. (**B**) The AA mutations in the subdomains S1 and S2 (SD1, SD2), N-terminal domain (NTD), and receptor binding domain (RBD) are highlighted.

To identify deleterious mutations in the SARS-CoV-2 genomes, we analyzed point-specific amino acid (AA) mutations in the genomes of this virus obtained from deceased COVID-19 patients using SIFT, PolyPhen-2, SNAP2, PROVEAN, PredictSNP, and MAPP web-based tools. Deleterious mutations were critically analyzed and cross-checked using these tools. A threshold value of − 2.5 was determined to ensure highly balanced accuracy in defining the deleterious mutation. Therefore, mutations having a value smaller than − 2.5 were identified as deleterious^33^. Among the AA mutations identified, the number of deleterious and non-deleterious mutations were 951 and 3199, respectively. The highest number of deleterious AA mutations were found in the ORF1b (n = 338) followed by ORF1a (n = 236), ORF3a (n = 122). Besides, 49, 45, 42, 40 and 30 deleterious AA mutations were predicted in the N, ORF8, ORF7a, S and ORF9b segments, respectively. In this study, the open reading frames (ORF) of the SARS-CoV-2 genome possessed a higher percentage of deleterious AA mutations than other segments. As for example, the ORF3a, ORF6, ORF7a, ORF8 and ORF9b harbored > 50.0% deleterious AA mutations. However, rest of the segments of the SARS-CoV-2 genomes fewer mutations (< 30) (Table S1). The overall AA mutations detected in the spike protein of the SARS-CoV-2 genomes of the  deceased patients are shown in Fig. 4A. In this study, the highest frequency of AA mutations (31.85%) was recorded in the *S* gene, which is responsible for viral pathogenicity. The *S* gene of the study genomes underwent AA mutations at 32 sites (Fig. 4A). Fourteen of these AA mutation sites such as V3L, L5Y, L10S, S13L, T19R, P26L, D401, S60A, P82AT, V1201Y204R, S2051, L2231, Y2651 were predicted in NTD (N-terminal domain) fragment, while eight of them (e.g., Q314, G339D, S371F, S373P, F377Y, D405N, K417N, L452R, T478K, and E484Q) were found in the RBD region. The remaining eight sites such as A570D, D614G, P681R, N764K, D796Y, N856K, R1000L, and E1188L were positioned in diverse areas of the S protein. The fusion peptide area was well conserved because no AA mutation hotspots were discovered (Fig. 4A).

Except for the significant AA mutation changes in the spike protein, there were notable changes in the mutational spectra of other proteins as well. In comparison to the different AA mutational spectra, a huge number of repeats were observed in the ORF1b (T67I, 2571 times) and N (G204R, 1484; R203K, 1559 times) segments. There were more seven AA mutations found to be occurred in > 500 sequences such as I2230T (543 times), A1708D (547 times) and T1001I (551 times) in ORF1a, L83F (631 times) in ORF3a, F3L (530 times), D34G (532 times) and F120L (532 times) in ORF8 fragment of the SARS-CoV-2 genome (Fig. 4B).

With the march of time, more and more deleterious AA mutations are being detected among the genome sequences of SARSCoV-2 especially those sequenced from the deceased COVID-19 patients. The top frequent AA mutations of different proteins occurring in different continents have been listed to better understand the scenario of SARS-CoV-2 mutational tendency depending on the regional factor (Table 2). The AA mutations occurring in more than two continents are highlighted to focus on them. Interestingly, D614G mutation in spike protein and S26L mutation in OF3a protein were found to occur in the SARS-CoV-2 genomes in all continents. Another noteworthy AA mutation, A1918V, occurred in both Asia and North American regions whereas a slightly different mutation, A1818L found in the African region. However, the ORF8 and ORF9b fragments showed no similarity of mutational alignment across the regional barrier (Table 2).

**Table 2:** Most frequent amino acid (AA) mutations predicted at various loci of SARS-CoV-2 genome obtained from deceased COVID-19  patients of the five continents.

Continent	Loci of SARS-CoV-2 genome									
	S	ORF1a	ORF1b	M	N	ORF3a	ORF7a	ORF7b	ORF8	ORF9b
ASIA	**D614G** P6081R A570D, **N501Y** **D405N**	P3142 S1188L A1708D **T265 I**	**P314L** **P1000L** **A1918V** **P10S**	**I82T** **A63T**	**G204R** **P13L, D3L** T205 I **D377Y**	**S26L** T223 I	82A **T120I**	**T40I**	L118V G8R	T60A A11V
NORTH AMERICA	**D614G** **T478K** G142D T1027 I S375F	V2930L A2710T P3395H **T3255 I**	**G662S** **A1918V** **R1315C** **I1566V** **P314L**	**I82T** **Q19E** **A63T**	S413R R203K R203M D63G **D377Y**	**S26L**	**T120I** **V82A**	V21F	V99L Y31F	G38V
EUROPE	**D614G** **L452R** **N501Y** K417T **D405N**	**T3255I** **P314L** **T3750I** G1307S	**P1000L** **G662S** **E1264D** **I1566V** **P10S**	**I82T** **Q19E** **D3G**	**P13L** R203K, D3L G215C P80R **G204R**	**T223I** **S26L**	**V82A**	L32F M1V	R101C A65G	P3H
AFRICA	**D614G** D950N E484K **N501Y** **P681H**	P1640L **T3750I** K1655N **T265I** T1001I	**P314L** **A1918L** R1315C **P314L** **E1264D**	**Q19E** **I82T**	**G204R** D63G S235F **D377Y** T205I	**S26L**	**T120I** **V82A**	**T40I**	G96S	G38R
SOUTH AMERICA	**D614G** **L452R** **P681H** **D405N** **T478K**	**T3255I** K1795Q K856R **P314L** S135R	**G662S** **P1000L** **E1264D** T2163I **R135C**	**A63T** **D3G**	S413R D3L G215C **D377Y**	**S26L** **T2231** ORF-3a	**T120I**	H42Y	T26I	L64F

Amino acid (AA) mutations occurred more than two continents are highlighted[bold].

### Effects of mutations on protein functions

**Figure 5:**
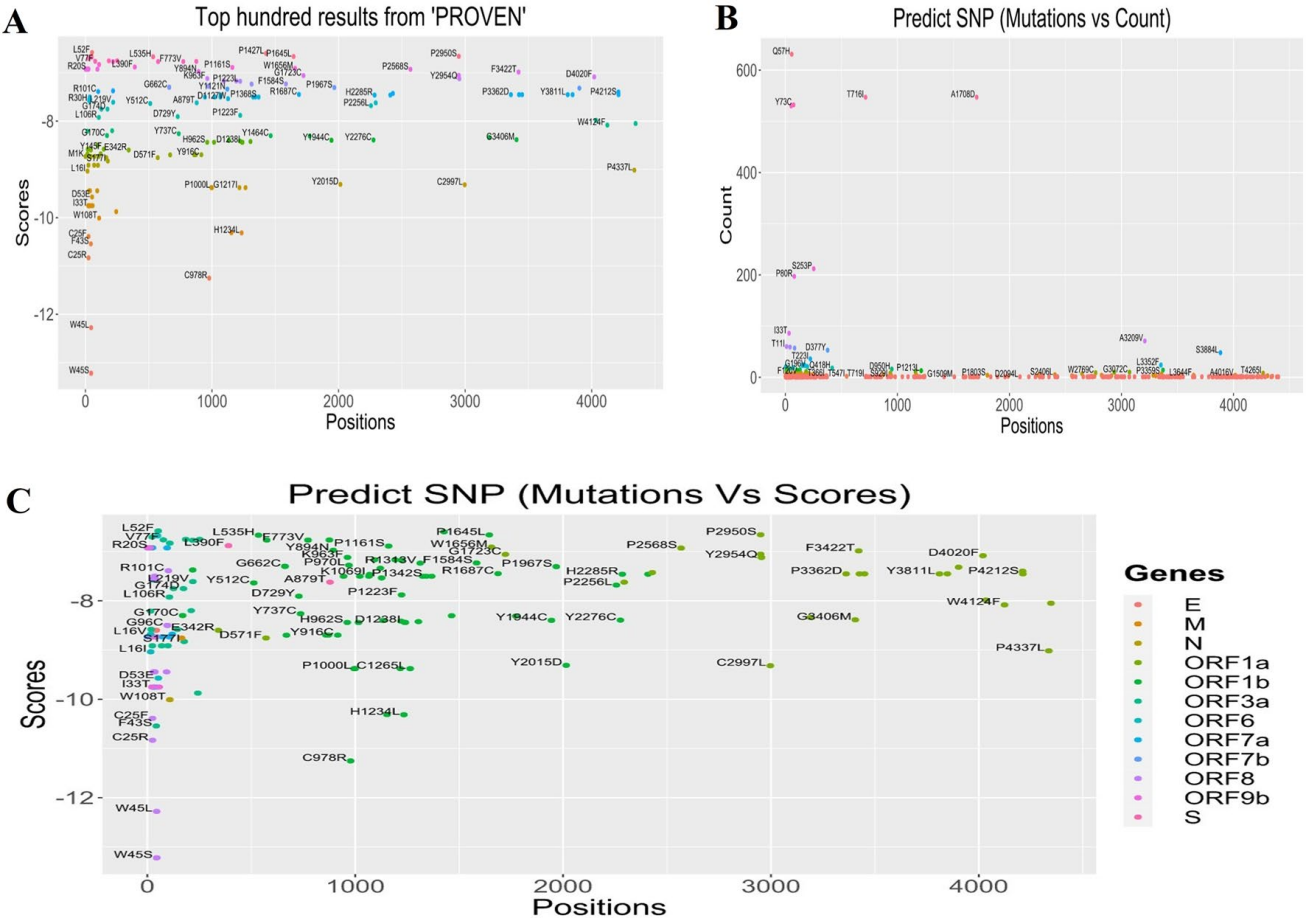
Scores of different mutations throughout the SARS-CoV-2 genomes sequenced from deceased COVID=19 patients. (**A**) Top hundred mutations predicted by PROVEAN tool. (**B**) Total frequency of the top mutations predicted by Predict SNP tool. (**C**) Prediction of deleterious mutations by Predict SNP.

Finally, we considered the deleterious signature mutations to evaluate the changes in proteins as biological functions using PROVEAN, PolyPhen-2, and Predict SNP tools (Fig. S1). We found the highest PROVEAN score of − 13.22 in case of W45S and W45R deleterious mutations, and a minimum − 12.278 for the W45L mutation of the *ORF8* gene (Fig. 5A). Interestingly, these three detrimental mutations were identified in the same ORF8 region of SARS-CoV-2 genomes using other tools. Using these three tools, G18V, W45S, I33T, P30L, and Q418H were identified as the frequent mutations which are responsible for defining each clade as they all are deleterious and unstable. Using Predict SNP, we simultaneously predicted the highest number of detrimental mutations (n = 1875) at Q57H in the *ORF3a* gene (Fig. 5B). Through the PolyPhen-2, we detected the highest number of deleterious mutations at D160Y (n = 1559) in the *M* gene and G204R (n = 1448) and D3L (n = 540) in the *N* gene. All these deleterious mutations had a PolyPhen-2 score of 1, whereas the sensitivity and specificity were 0 and 1, respectively. These findings indicate that differences in mutations in distinct regions will likely impact protein function. Top mutations against the Predict SNP score are visualized in Fig. 5C. The mutations occurred in the ORF8 segment such as W45L andW45S scored the most negative values according to Predict SNP prediction model where both scored less than -12. No other mutation of this segment or other proteins had scored such negative scores throughout the mutational spectra (Fig. 5C).

## Discussion

Analyzing mutations in SARS-CoV-2 genomes presents a valuable opportunity to gain insights into how various genes undergo frequent changes that can influence viral characteristics and disease manifestation. One of these viral features, the transmissibility of the SARS-CoV-2 virus, is impacted by genetic variations within the virus genome. Despite these genetic variations, there’s an ongoing need to develop new vaccines that can adapt to emerging variants^34^. In this study, we examined the mutational patterns in SARS-CoV-2 genomes from deceased COVID-19 patients across five continents. For the first time, we have identified both nucleotide (NT) and amino acid (AA) mutations, including both deleterious and non-deleterious changes, in the SARS-CoV-2 genomes of deceased COVID-19 patients on a global scale. We discuss the implications of these mutations for genomic surveillance and the management of this viral disease. Additionally, we present the epidemiological distribution of different variants and clades in the SARS-CoV-2 genomes of deceased patients using a robust phylogenetic approach.

Genome-wide variations in SARS-CoV-2 reveal evolution and transmission dynamics of several variants which are critical considerations for control and prevention of COVID-19. The SARS-CoV-2 pandemic has led to over 6.9 million deaths as of October 2023 (https://www.worldometers.info/coronavirus/).  We analyzed 5,724 full-length genomes of the SARS-CoV-2 (after thorough filtering of 243,270 genomes) sequenced from deceased COVID-19 patients of diverse demographics from January 2020 to February 2023, and detected some shared deleterious mutations associated with the morbidity of COVID-19 patients in different continents. Furthermore, we traced the spread of the variants worldwide and provided an insight into the evolutionary pressure in SARS-CoV-2 genomes associated with its virulence.

In the current study, we applied the open-source programs GISAID EpiCoV™ Database^35^ and Nextstrain^36^. By comparing the death severity rates of SARS-CoV-2 infections in 123 countries and five continents across the globe, we found that the highest amount (> 33.0%) of SARS-CoV-2 genomes from deceased COVID-19 people were sequenced from North America followed by Europe (~ 29.0%), South America (~ 20%), and Africa (~ 18%). However, the lowest number of SARS-CoV-2 genomes from deceased COVID-19 patients were sequenced from Asian countries (< 1.0%). The country-wise distribution of these genomes in top ten countries also revealed significant discrimination showing that Brazil (> 16.0%), Mexico and USA (> 14.0%, each), Bulgaria (> 13.0%) and India (> 5.0%) are the countries from where higher number of genomes were sequenced from deceased COVID-19 people. However, the lowest amount of WGS was sequenced in one transcontinental North American country, the Panama (0.5%). In addition, male COVID-19 patients having an average age of 55 years had higher COVID-19 risk than female patients (average age of 70 years). The genetic differences among SARS-CoV-2 strains could be linked with their geographical distributions and COVID-19 severity. The findings of the present study are in line with several earlier researches that reported that the integration of geographical and climatic data with genetic mutation analysis promises to provide a fuller understanding of the origins, dispersal and dynamics of the evolving SARS-CoV-2 virus^2, 5, 37^. Since the emergence of the pandemic in China in December 2019, thousands of variants of SARS-CoV-2 have emerged^38, 39^. The SARS-CoV-2 genomes sequenced from deceased people belonged to 21 Nextstrain clades comprising both VOCs (e.g., 20I, 20H, 20J. 21A, 21I, and 21J) and VOIs (e.g., 21C, 21G and 21H). This virus is continuously mutating from its ancestral strain (e.g., hCoV-19/Wuhan/WIV04/2019), which results in the upsurge of new variants. One of the interesting findings of this study is that clade 20I (Alpha variant) was the most predominating clade followed clade 20H (Beta variant) and clade 20J (Gamma variant) in the SARS-CoV-2 genomes of deceased COVID-19 patients. These results are consistent with previous studies that reported Alpha variant as the most prevailing VOC followed by Beta and Gamma VOCs^40, 41^. Currently, multiple variants are circulating globally^39, 41^. Recently, there have been five VOC that drew tremendous public attention due to increased transmissibility or virulence that may attenuate the effectiveness of current control measures, available diagnostics, vaccines, and therapeutics. SARS-CoV-2 is highly probable to mutate and evolve to enhance its infectivity and transmissibility, posing a severe risk of accumulation and dominance of immunologically relevant mutations across different lineages in the near future. Accumulation of single or multiple mutations at the RBD-ACE2 interface can lead to more deadly waves of COVID-19. A thorough inspection of hot-spot residues, genomic epidemiology, evolutionary history, and selective pressures can help to predict new mutations.

Through an in-depth investigation into the emergence and pursuit of VOCs, such as Alpha, Beta, Gamma, and Delta, and VOIs, such as Eta, and several Nextstrain clades, this study reports the emergence and spread different variants, clades and/or lineages of the SARS-CoV-2 in deceased COVID-19 people from across the globe. Our analysis showed that B.1.1.529 (Omicron), B.1.617.2 (Delta), B.1.1.7 (Alpha), B.1.1.28 (Brazilian variant), P.1 (Gamma), and B.1.1.519 (Mexican variant) were the most prevalent variants/lineages in the genomes of the SARS-CoV-2 genomes of the deceased patients. One of the key findings of this study is the prevalence of the 'G' clade in the SARS-CoV-2 genomes of deceased COVID-19 patients in Asian, African, and North American regions. In contrast, most of the death cases in Europe and South America were associated with the 'GRY' and 'GR' clades, respectively. Due to a significant antigenic shift, the SARS-CoV-2 virus has shown extensive mutations that have resulted in some VOCs, including Alpha, Beta, Gamma, Delta, and Omicron. There are ample researches investigating the transformation in the SARS-CoV-2 genomes^2, 42, 43^. However, they do not specifically study the mutations in SARS-CoV-2 genome sequences retrieved from deceased COVID-19 individuals.

Out of 5,724 complete genomes of SARS-CoV-2 extracted from deceased COVID-19 patients, we detected 35,799 NT mutations, with the majority (31.8%) occurring on the spike (S) protein. The *S* gene underwent NT mutations at 32 sites including 14 and eight mutations in the NTD and RBD regions, respectively, and rest eight mutations were detected other sites of the S protein. In addition, ORF1b, N, ORF1a, and ORF8 fragment of the SARS-CoV-2 genome also underwent to NT mutations at different positions. A mutation is a natural aspect of viral reproduction during disease outbreaks, with RNA viruses exhibiting more mutations than DNA viruses^44^. Irrespective of their impact on viral viability, the mutations found in the genome of these viral progenitors will dominate the population. This interaction between natural selection and serendipity influences the evolution of viruses within individuals, groups, and regions^45, 46^. The D614G sites were determined to have the most mutations changing NT patterns. In this study, the D614G mutation was predominant in deceased people in 10 countries, such as Brazil, Mexico, USA, India, Iran, China, the UK, France, Japan, and Germany, and five continents. The D614G mutation was observed to increase viral fitness and infectivity^47, 48^. Several researchers have reported the functional significance of D614G in the S protein, linking its role to the increased pathogenesis of the virus^49^. The spike protein change at D614G was dominant throughout the world, with increased infectivity and transmission^37^. Moreover, mutations in the spike proteins concurrently elevate the viral attachment to ACE2 receptors in the cell surface of the host cell^50^. In addition, most countries evidenced D614G mutations compared to the other sites^47, 48^. However, existing evidence is inconclusive about the sole effect of the D614G mutation on pathogenicity and fatality, as numerous issues, such as aging and comorbidity, play a crucial influence^44^. One of the important findings of this study is the estimation of predominating “G” clade in the Asian, African and North American regions while most of the death cases in Europe were registered with “GRY” clade. Different clades of the SARS-CoV2 have been identified during the pandemic. Some spread worldwide, while others quickly faded away. Identification of the circulation of clades and/or variants in a region/country/society is important for better understanding of circulation of different clades/variants, genetic diversity and mutations in all non-structural, structural and accessory genes^51^.

One of the standout findings of this study is the discovery of 4,150 AA mutations, encompassing both deleterious and non-deleterious changes, distributed across various segments of the SARS-CoV-2 genomes. These mutations exhibited significant variations among the genomes of the deceased individuals from five different continents. Most of the ORFs of the SARS-CoV-2 genomes possessed higher deleterious AA mutations than other segments of the genomes. Our findings confirmed that the D614G mutation in the spike protein and the S26L mutation in the ORF3a fragment were present in the SARS-CoV-2 genomes from the deceased patients of all continents. Our results corroborate with many of the previous researches which reported that the spike gene mutations account for most of the clinically influential VOCs while the ORF1a frame of the genome serves as a key region for NSP (non-structural proteins) mutations^2, 52^. Since the first report of the D614G mutation in SARS-CoV-2 genomes^53^, other modifications have also been reported^2, 23, 48^. This finding put forward that these two mutations might have been associated with increased viral transmission and infectivity and have likely arisen independently in multiple regions, indicating that they may have a selective advantage. The persistence of these mutations across different populations underscores the importance of monitoring the mutational tendencies of the virus to inform public health responses and the development of effective treatments and vaccines. Although vaccination reduces symptomatic cases, hospitalizations, and mortality, the rapid mutation of the virus and the emergence of new variants (e.g., VOIs or VOCs) limit the effectiveness of these vaccines^54^. One of the major concerns about these emerging mutations is that they could potentially lead to dangerous modification in the SARS-CoV-2 genome that would ultimately increase infection severity or a failure of the currently developed ‘vaccines' effects. Moreover, the detected VOIs or VOCs may make vaccinated persons to be re-infected with new-fangled variants^54^. However, scientists across the globe are trying to develop and formulate new vaccines (or vaccine candidates) against the newly emerged VOIs or VOCs of SARS-CoV-2. The updated vaccines are not expected to prevent all cases of COVID-19, rather they may reduce severe illness, hospitalization, and death from infection^55^.

Another novel aspect of our study is that the findings of the present study identified the regional differences in the AA mutation patterns of the SARS-CoV-2. For instance, the A1918V mutation was identified in the SARS-CoV-2 genomes of COVID-19 deceased people of both Asian and North American regions, while a slightly different A1818L mutation was present only in African region suggesting that different population genetics and transmission patterns may be driving these differences. These regions of the virus may be more conserved due to functional constraints, and mutations in these regions may have a greater impact on the viral pathogenesis. Remarkably, we found that there was a lack of similarity in mutational spectra between the ORF8 and ORF9b fragments of the virus across the regional barrier. These regions of the SARS-CoV-2 genome might be more conserved due to functional constraints, and mutations in these regions may have a greater impact on the virus pathogenesis. Other common mutations identified in this study included R203K and G204R, containing two AA alterations in N protein due to a trinucleotide substitution. R203K was detected primarily in samples from Mexico, Iran, the United Kingdom, and other regions across North America and Europe. On the other hand, AA mutation G204R was found predominantly in samples from India, Spain, France, and Germany and across the continents of Asia, Africa, and Europe. The AA changes in R203K and G204R were anticipated to reduce protein stability. This finding is line with other researches that identified R203K and G204R, which destabilize the structure of the N protein while improving interaction with the Envelope protein to enhance viral release^5, 56^. The N protein contributes to the creation of spiral ribonucleoproteins during RNA genome packing, thus influencing viral reproduction and altering the homeostasis of infected individuals^57^. Modifications in the viral N protein structure enhanced its replication, pathogenicity, and adaptability^56^. We also found that AA changes patterns are different throughout the SARS-CoV-2 genomes. The high proportion of deleterious AA mutations were predicted in the open-reading frames than other segments. The high morbidity and mortality of immune-compromised patients infected by SARS-CoV-2 might be correlated with the disastrous consequences of these deleterious AA mutations^2^. In addition, several deleterious mutations manifest as geographic patterns alluding to the virus ability to modify/adapt itself within a distinct microenvironment. To acquire comprehension of these mutation patterns, it is important to have insights into their respective mutation frequency across both local and global patterns and help preempt the emergence of VOIs and VOCs especially in COVID-19 deceased patients. The COVID-19 pandemic with these VOIs and VOCs have exacerbated inequality within and between countries, eroded global solidarity and trust, and caused dramatic backsliding on key health outcomes due to disrupted access to essential health services. SARS-CoV-2 carrying several AA mutations throughout the genome results in infectivity of the COVID-19 disease in humans and thus accumulating mutations over time has become a public health concern globally^33, 58^. The findings of this study emphasize that with each AA mutation in the structural segment of SARS-CoV-2, the evolved variants of SARS-CoV-2 might have acted differently and became more lethal leading to an increased in case fatalities. Moreover, it is a realistic possibility that over time VOIs and VOCs of SARS-CoV-2 have emerged, creating an alarming situation globally that necessitated the urgency of developing a vaccine to mitigate the SARS-CoV-2 impact on public health, the economy and society.

It's important to note that this study has some limitations, primarily stemming from the relatively small sample size and the potential for sampling bias. The study was constrained by the availability of a limited number of complete SARS-CoV-2 genomes, with approximately 5700 genomes obtained from deceased COVID-19 patients around the world. Additionally, there was a lack of data normalization. These limitations are partly due to the fact that not all countries worldwide sequenced SARS-CoV-2 genomes from all deceased COVID-19 cases or uploaded them to public databases like GISAID. For instance, the top five contributing countries in this study were Brazil (n = 456), USA (n = 419), Bulgaria (n = 361), India (n = 175), and France (n = 115). Furthermore, as the number of SARS-CoV-2 genomes evolves over time, we focused on identifying point-specific mutations in the SARS-CoV-2 genomes of deceased COVID-19 patients. Therefore, the mutation patterns should be considered as approximate findings. It's also worth noting that due to variability in different normalization methods, it's not appropriate to directly compare data between two or more structural segments of the SARS-CoV-2 genome, as a value for one segment may not have the same significance as the same value for another segment. This study opens up new perspectives for determining whether these frequent mutations might lead to biological differences and exploring their correlation with varying case fatality rates or COVID-19-related deaths.

## Conclusion

This study focused on identifying mutational patterns and their roles in SARS-CoV-2 genomes from deceased COVID-19 patients across diverse continents, uncovering shared deleterious mutations. The research revealed 21 Nextstrain clades within SARS-CoV-2 genomes, including four variants of concern (VOC), namely 20I (Alpha), 20H (Beta), 20J (Gamma), and 21A, 21I, and 21J (Delta), found in specimens from five continents. Notably, there was significant heterogeneity in the distribution of SARS-CoV-2 genomes, with Brazil contributing the highest proportion (over 16.0%) of sequenced SARS-CoV-2 genomes from deceased individuals to the GISAID database. It was observed that the 'G' clade was predominant in the Asian, African, and North American regions, while the 'GRY' clade was predominant in Europe. The spike protein underwent the highest number of NT and AA mutations, with two common (e.g., D614G and N501Y) deleterious AA mutations in five continents. Although, G18V, W45S, I33T, P30L, and Q418H mutations were identified as the most frequent clade defining deleterious mutations, the highest number of detrimental mutations at Q57H was detected in the *ORF3a* gene. This study offers new perspectives to determine whether one of these frequent mutations would lead to biological differences, and their correlation with different case fatality rates. The mutations identified in this study throughout the SARS-CoV-2 genomes sequenced from deceased COVID-19 patients may help us in understanding its diversification, volatility, and molecular etiology better, which could offer an opportunity to produce efficient and safe therapeutics and vaccines for this highly changeable coronavirus. The variation in mutational patterns between demographic groups may indicate the rate of infection, mortality, and vaccine effectiveness. Hence, the findings of this study bear significant implications for genomic surveillance and the development of effective preventive and therapeutic strategies for managing the COVID-19 pandemic. Therefore, future research with larger sample sizes, irrespective of SARS-CoV-2 variants, clades, or lineages, should be conducted at the individual level. This should encompass analyses of metabolic functional pathways, intra-viral processes, and virus-host interactions to gain a comprehensive understanding of how these mutations impact the immunity of distinct ethnic groups.

## Materials and methods

### Ethical clearance

No ethical approval was required in this in silico study.

### Genomic data collection and filtering

To decipher the mutational spectra and their consequences in SARS-CoV-2 genomes of the deceased COVID-19 people, 243,270 SARS-CoV-2 complete whole genome sequences (WGS) were retrieved from the global initiative on sharing all influenza data (GISAID) (https://www.gisaid.org/) EpiCoV™ Database^35^ from January 2020 to February 2023 (Data S1). We filtered these sequences based on some specific criteria (e.g., full-length WGS, high read coverage, deceased COVID-19 people, collection date). Only complete genomes with a size of > 29,000 bp were selected, and those with low range, i.e., possessing > 5% of N’s, were filtered out. After thorough filtering, 5724 WGS of SARS-CoV-2 were retained, and selected finally for this study. Pyfasta (http://github.com/brentp/pyfasta/) split the retrieved sequences into smaller files for further analysis. All the metadata (including submission date, strain, GISAID ID number, region/location, division, length, host, age, sex, originating and submitting laboratory, and authors) from GISAID were collected for statistical analysis (Data S1).

### Phylogenetic analysis

The Nextstrain (https://clades.nextstrain.org/) platform was utilized for the analysis related to phylogeny and mutations of selected SARS-CoV-2 complete genomes (Hadfield et al.^36^). Nextclade Web 2.14.1 (https://clades.nextstrain.org/results; accessed on October 10, 2023) was used to check the sequence quality, pairwise sequence alignment, and generate the designated clade tree. The inferred evolutionary relationship of the study genomes obtained from deceased COVID-19 patients was built and visualized using the Nextstrain, applying phylogenies, entropy, geographic maps, and frequency graphs. The initial phylogenetic tree was constructed using IQTREE v1.6.12^59^, which implements the GTR (generalized time-reversible) model and bootstraps the tree topology to ensure a high degree of confidence. The bare tree was rooted using the reference genome Wuhan/Hu-1/2019 (Retrieved from https://www.epicov.org with GISAID ID: EPI ISL 402125). The tree was further processed using TreeTime v0.8.1^60^ to generate a time-resolved phylogeny based on maximum likelihood. The Auspice web server (https://auspice.us/) was used to visualize the tree. Mutations and clades were assigned from the CoVserver mutation app of the GISAID (https://gisaid.org/database-features/covsurver-mutations-app/) where hCoV-19/Wuhan/WIV04/2019 (GenBank accession no. MN996528.1) was used as a reference genome^42^.

### Mutation profiling in SARS-CoV-2 genomes and prediction of their effect on protein function

Mutations were defined as AA substitutions using the hCoV-19/Wuhan/WIV04/2019 isolate as the reference sequence. The filtered SARS-CoV-2 complete genomes were aligned against the reference genome (hCoV-19/Wuhan/WIV04/2019) by MAFFT v7.470^61^ to predict the frequency of occurrence of each mutation. The aligned sequences containing no nucleotide ambiguities were imported into and analyzed in different web-based tools to predict mutations. These tools included Sorting Intolerant from Tolerant (SIFT) (http://sift.jcvi.org/), Polymorphism phenotyping-2 (PolyPhen-2; http://genetics.bwh.harvard.edu/pp2), Screening of Nonacceptable Polymorphism 2 (SNAP2; https://www.rostlab.org/services/SNAP/), Protein Variation Effect Analyzer (PROVEAN; http://provean.jcvi.org/index.php), PredictSNP (https://loschmidt.chemi.muni.cz/predictsnp/), and Multivariate Analysis of Protein Polymorphism (MAPP; http://www.ngrl.org.uk/Manchester/page/mapp-multivariate-analysis-protein-polymorphism.html). SIFT is a standalone version of the web server that predicts the potential impact of AA substitutions on protein function^62^. The PolyPhen-2 is a tool that predicts the possible impact of AA substitutions on the human protein structure and function using structural and comparative evolutionary considerations^63^. The mutation score for PolyPhen-2 is between 1 (harmful) and 0 (neutral)^63^. Likewise, SNAP2 is a bioinformatics tool that uses the annotations from the protein mutant database (PMD) to predict the changes due to the non-synonymous single nucleotide polymorphisms (nsSNPs) on protein function^64^. The PROVEAN tool generates a score for each mutation to predict its impact on host cells, whether harmful or neutral. The mutation score exceeding the default threshold of -2.5 suggests a neutral effect, whereas a score below the threshold indicates a detrimental effect^65^. The MAPP method predicts the deleteriousness of non-synonymous SNPs through an alignment of interspecific sequences that are putatively orthologous to the protein of interest^66^. To predict the overall effect of AA mutations on protein function (topology and flexibility), PredictSNP^67^, PolyPhen-2^63^, and PROVEAN^65^ were utilized. PredictSNP is a consensus classifier combining the datasets from SIFT, PolyPhen-2, SNAP, PROVEAN and MAPP. This consensus classifier gives significantly improved and accurate predictions over the individual tool. Deleterious mutations are critically analyzed and cross-checked using these tools. A threshold value of − 2.5 was determined to ensure highly balanced accuracy in defining the deleterious mutation. Therefore, mutations having a value smaller than − 2.5 were identified as deleterious^33^. The specificity and sensitivity values allude to confidence in the prediction (Fig. S1).

### Statistical analysis

The statistical analysis was performed using the R statistical environment package v 3.6.3 (https://cran.r-project.org/bin/windows/base/old/3.6.3/). Categorical variables were expressed as absolute frequency and percentages. The different single nucleotide variation (SNV) counts per genome between different countries and/or continent was analyzed using one-way ANOVA followed by the Chi-square test. The Chi-square test assessed the relationship between each mutation and patient status. False discovery rate (FDR) was calculated using the Benjamini–Hochberg method to accommodate multiple hypothesis testing, and only results exceeding an FDR cut-off value of 5% were considered significant. All *p*-values were calculated from two-sided tests using 0.05 as the significance level.

### Supplementary Information

Supplementary Information 1.

Supplementary Information 2.

## Data Availability

The SARS-CoV-2 genome sequence data used in this study were retrieved from the GISAID Initiative (https://gisaid.org). Information on all of the SARS-CoV-2 sequences used in this manuscript are available in the supplementary information file (i.e., data availability statement).
