# Supplementary material for: Some common deleterious mutations are shared in SARS-CoV-2 genomes from deceased COVID-19 patients across continents
Source: Sci Rep. 2023 Oct 30;13:18644. doi: 10.1038/s41598-023-45517-1 (PMC10616235; doi:10.1038/s41598-023-45517-1)
Supplement: Supplementary file 1 — Supplementary Information 1. [file 41598_2023_45517_MOESM1_ESM.pdf]

Supplementary Information

Table S1. Amino acid mutations (**deleterious and non- deleterious**) predicted at various loci of SARS-CoV-2 genomes extracted from dead deceased people of five continents.

Fragment	Deleterious	Non-Deleterious	Percentage of Del Mutation
E	8	12	40.00%
M	8	34	19.05%
N	49	263	15.71%
ORF1a	236	1686	12.28%
ORF1b	338	485	41.07%
ORF3a	122	114	51.69%
ORF6	23	12	65.71%
ORF7a	42	39	51.85%
ORF7b	10	21	32.26%
ORF8	45	40	52.94%
ORF9b	30	26	53.57%
S	40	467	7.89%

Figure

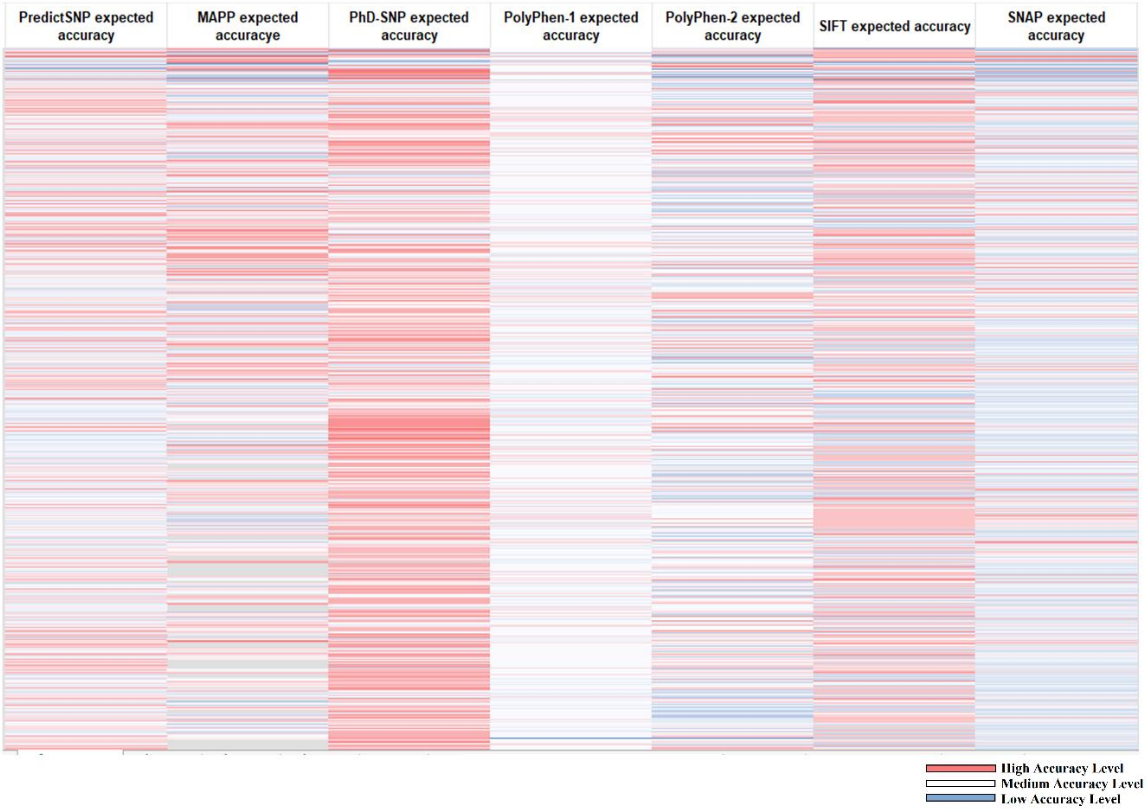

Fig. S1: Accuracy of predicting mutations by different tools.

Data Availability Statement

GISAID Identifier: EPI_SET_230306ke
DOI: 10.55876/gis8.230306ke
All genome sequences and associated metadata in this dataset are published in GISAID's EpiCoV database. To view the contributors of each individual sequence with details such as accession number, Virus name, Collection date, Originating Lab and Submitting Lab and the list of Authors, visit 10.55876/gis8.230306ke

Data Snapshot

- EPI_SET_230306ke is composed of 243,270 individual genome sequences.
- The collection dates range from 2020-01-08 to 2023-02-21;
- Data were collected in 158 countries and territories;
- All sequences in this dataset are compared relative to hCoV-19/Wuhan/WIV04/2019 (WIV04), the official reference sequence employed by GISAID (EPI_ISL_402124). Learn more at https://gisaid.org/WIV04.
